# Atypical presentation of placental abruption

**DOI:** 10.11604/pamj.2020.36.70.23441

**Published:** 2020-06-08

**Authors:** Mounir Moukit, Jaouad Kouach

**Affiliations:** 1 Department of Obstetrics and Gynecology, Military Training Hospital Mohammed V, Hay Riyad, 10100, Rabat, Morocco,; 2 Faculty of Medicine and Pharmacy, University Mohammed V, Rabat, Morocco

**Keywords:** Threatened preterm labor, placental abruption, ultrasound

## Image in medicine

A 25-year-old primigravida with no significant medical history was referred to our department, at 30 weeks´ gestation, for threatened preterm labor. On general examination, the patient was afebrile with normal vital signs. Obstetrical examination revealed fundal height corresponding to gestational age and regular uterine contractions without uterine rigidity. Fetal heart rate was normal. On speculum, the cervix was macroscopically normal without bleeding, amniotic fluid or vaginal discharge. Vaginal examination objectified an open cervix (2cm). Ultrasound scan showed a single live intrauterine fetus in cephalic presentation of 29 - 30 weeks with oligoamnios. Placenta was anterior and thick with a hyper echoic basal area measuring 8cm × 3cm suggestive of placental abruption. Her blood investigations were normal. Under spinal anesthesia, an emergent caesarean section was performed giving birth to a male newborn, weighing 1200g with normal Apgar scores. Macroscopic examination of the placenta confirmed the retro placental clot in maternal surface. The postoperative period was uneventful for patient and the newborn was transferred to neonatal intensive care unit. The highly variable presentation of placental abruption makes the clinical diagnosis difficult. As was in the present case, it should be suspected in any case of unexplained threatened preterm labor. Ultrasound evaluation can play a role in the diagnosis of acute placental abruption especially in those with atypical clinical presentation.

**Figure 1:**
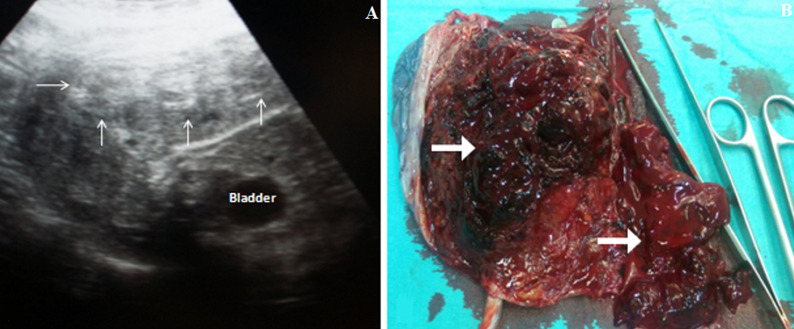
A) obstetrical ultrasound objectified the retroplacental hematomas (white arrows); B) macroscopic view of the adherent blood clot on maternal surface (white arrows)

